# Change and Variability in East Antarctic Sea Ice Seasonality, 1979/80–2009/10

**DOI:** 10.1371/journal.pone.0064756

**Published:** 2013-05-21

**Authors:** Robert Massom, Philip Reid, Sharon Stammerjohn, Ben Raymond, Alexander Fraser, Shuki Ushio

**Affiliations:** 1 Australian Antarctic Division, Department of Sustainability, Environment, Water, Population and Communities, Kingston, Tasmania, Australia; 2 Antarctic Climate and Ecosystems Cooperative Research Centre, University of Tasmania, Sandy Bay, Tasmania, Australia; 3 Australian Bureau of Meteorology, Centre for Australian Weather and Climate Research, Hobart, Tasmania, Australia; 4 Institute of Arctic and Alpine Research, University of Colorado, Boulder, Colorado, United States of America; 5 National Institute of Polar Research, Tachikawa, Tokyo, Japan; University of Tasmania, Australia

## Abstract

Recent analyses have shown that significant changes have occurred in patterns of sea ice seasonality in West Antarctica since 1979, with wide-ranging climatic, biological and biogeochemical consequences. Here, we provide the first detailed report on long-term change and variability in annual timings of sea ice advance, retreat and resultant ice season duration in East Antarctica. These were calculated from satellite-derived ice concentration data for the period 1979/80 to 2009/10. The pattern of change in sea ice seasonality off East Antarctica comprises mixed signals on regional to local scales, with pockets of strongly positive and negative trends occurring in near juxtaposition in certain regions e.g., Prydz Bay. This pattern strongly reflects change and variability in different elements of the marine “icescape”, including fast ice, polynyas and the marginal ice zone. A trend towards shorter sea-ice duration (of 1 to 3 days per annum) occurs in fairly isolated pockets in the outer pack from∼95–110°E, and in various near-coastal areas that include an area of particularly strong and persistent change near Australia's Davis Station and between the Amery and West Ice Shelves. These areas are largely associated with coastal polynyas that are important as sites of enhanced sea ice production/melt. Areas of positive trend in ice season duration are more extensive, and include an extensive zone from 160–170°E (i.e., the western Ross Sea sector) and the near-coastal zone between 40–100°E. The East Antarctic pattern is considerably more complex than the well-documented trends in West Antarctica e.g., in the Antarctic Peninsula-Bellingshausen Sea and western Ross Sea sectors.

## Introduction

Better identification, quantification and understanding of change and variability in global sea ice coverage are increasingly recognised as a high priority in climate and ecological research. Sea ice plays a crucially-important role as a key modulator of the Earth's climate system, a sensitive bellwether of climate variability/change ([1], and references therein), and a critical habitat [2]. As such, changes to the seasonality of sea ice coverage have important and wide-ranging implications. This sense of urgency has been heightened by recent observations of strong regional changes in both the areal extent [3]–[4] and seasonality of sea ice distribution in both the Arctic and western Antarctic [5]–[6], and resultant concern over possible associated complex seasonal feedbacks and non-linear processes that may drive further change e.g., [6]–[7].

Seasonality here collectively describes the timings of annual sea ice advance and retreat and resultant duration at any given location – as opposed to sea ice extent, which is a descriptor of the area of ocean covered by sea ice above a threshold concentration. As stated in [8], the distinction between sea ice extent and seasonality is an important one, and for a number of reasons. Seasonal open-water duration (ice-free summer length) controls solar heating and wind-mixing of the upper ocean [9]–[10], to affect sea-surface temperatures [11] and ocean upwelling [12]. Moreover, high-latitude ecosystems are specifically adapted to both the presence and seasonal rhythms of sea ice [2].

In this paper, we carry out the first detailed analysis of spatio-temporal patterns of change and variability in sea ice seasonality in East Antarctica, over the period 1979/80–2010/11 based on daily satellite ice concentration data. To date, Antarctic work assessing seasonality change has largely focused on the western hemisphere, where major change has been identified in the western Ross Sea (∼2 month shortening of the summer ice-free season since 1979/80) and the Antarctic Peninsula and Bellingshausen Sea region (>3 month lengthening of open water conditions). The magnitude of the latter is in fact even greater than the loss that has occurred in regions of greatest decline in the Arctic i.e., the western Beaufort, East Siberian and Chukchi seas [6].

By contrast, relatively little is known about patterns of change and variability in sea ice seasonality across East Antarctica, where sea ice coverage is strongly (though not exclusively) seasonal and occurs in a relatively narrow yet complex zone. This represents a major knowledge gap, and one that has severely compromised our ability to gauge and understand circum-Antarctic climate change and variability and their biological and physical impacts. The crucial need for improved knowledge of change and variability in East Antarctic sea ice seasonality is underpinned by the fact that seasonal loss, or alternatively gain, of sea ice in this region can have global consequences, given that the coupling between sea ice, oceanic and atmospheric circulation and temperature and biogeochemical cycles can result in positive feedbacks that drive climate change [13]. Changing seasonal coverage of sea ice may also impact the strength of ocean overturning circulation via its effect on the ocean freshwater balance [13]. In addition, observations from the Western Antarctic Peninsula region also suggest that the longer ice-free summer there and increased westerly winds drive greater wind mixing and upwelling of warm Circumpolar Deep Water onto the continental shelf [12], to increase ocean heat content from below [14]. This can in turn lead to enhanced basal melt of floating ice-sheet margins [15]–[16].

Biological ramifications of change in East Antarctic sea ice seasonality are also likely to be profound [17]. A major concern is that possible changes in the timing of sea ice advance and retreat in East Antarctica could also lead to major (but as yet undetermined) changes in habitat, food type and availability, species distributions and thus ecosystem dynamics and biogeochemical cycling in that region. This is predicated by strong evidence from the West Antarctic Peninsula region that the recent shortening of the sea ice season there [6], [18]–[19], is having dramatic impacts across multiple levels of the marine ecosystem via disruption of key phenological relationships e.g., [10], [20]–[21]. In addition, changing patterns of sea ice seasonal growth and decay have implications for the biogeochemical cycling and air-sea exchange of climate gases such as CO_2_ and thus potentially ocean acidification [22]–[25].

## Data and Methods

Maps of annual days of advance and retreat, and resultant ice season duration, for the sector 30–170°E were computed from daily sea ice concentration data obtained from the US National Snow and Ice Data Centre (http://nsidc.org). The dataset used is the NASA Bootstrap SMMR-SSM/I combined dataset (http://nsidc.org/data/docs/daac/nsidc0079_bootstrap_seaice.gd.html), which offers complete coverage of the Antarctic sea ice zone on a daily basis after July 1987 and every other day prior to that (back to October 1978), at a spatial resolution of 25×25 km. Following [18], after [5] and [26], annual maps of patterns of ice advance and duration were derived by flagging the timings of the advance and retreat of the ice edge within an annual search window that begins and ends during mean summer (mid-February) minimum ice extent (i.e., year day 46 to 410, or 411 in leap years). Within this period, annual day of advance is the time when the ice concentration in a given pixel first exceeds 15% (taken to approximate the ice edge) for at least 5 days, while day of retreat is the time when concentration remains below 15% until the end of the given sea ice year. Ice season duration is then the period between day of advance and retreat. For regions where ice remains (survives the summer melt), day of advance and retreat are set to the lower and upper limits, respectively i.e., year day 46 and 410/11. Isolated days of missing data were interpolated from adjoining days. Larger gaps during December 1987 through mid January 1988 were filled with the 1979–2009 climatology.

The analyses and presentation of results follows this general progression:

- Identification of mean patterns of advance, retreat, duration.
- Analysis of correlations of mean annual patterns of advance and retreat versus duration, to determine whether variability in advance or retreat is the stronger determinant of ice season duration.
- Investigation of interannual variability in these patterns, in two ways. Firstly, for selected seasons (1980/81, 1999/2000 and 2004/05), anomalies in advance, retreat and season length were calculated relative to the long-term means for 1979/80–2009/10. Variability was also assessed more generally (i.e. across all years) in terms of the standard deviation in day of advance/retreat and ice season duration.
- Analysis of change and variability in ice conditions (concentration) along the 110°E and 140°E meridian. These were chosen in order to examine seasonality variability within a given ice regime, and as they are long-term biological monitoring transects for Australia and Japan i.e. to provide physical input to studies analysing biological change and variability. Two transects at 90°E and 100°E were also examined for comparison, in that they intersected regions that exhibited different sea ice seasonality patterns. Taken together
- Calculation of trends in sea ice seasonality over the entire time series (1979/80–2009/10). These form the basis of identification and detailed analysis of spatio-temporal patterns of sea ice change, including identification of sea ice-mediated “hot-spots” (i.e., unusually large or persistent areas of change), on the assumption that the trends are linear.

Information on major features of oceanic circulation across East Antarctica, for comparison with observed climatological (mean) patterns of sea ice seasonality derived by this study, was obtained from [27] and [28]. Associated bathymetric data was obtained from the ETOPO1 dataset [29]. Attribution of factors responsible for observed patterns of change and variability in sea ice seasonality is beyond the scope of this study. However, the new results from the sea ice analyses are compared to sea surface temperature (SST) trends off the ice edge. The SST data are those of Reynolds and Smith OI.v2, which are available from late 1981 onwards [30].

Unless otherwise specified, “sea ice” refers here to the moving pack ice and stationary landfast sea ice (fast ice) combined. Fast ice forms a narrow zone (typically<100 km) around the Antarctic coastal margin, where its distribution is associated with (and is governed by) coastal promontories, sheltered embayments and groups of icebergs grounded in waters shallower than approximately 350–400 m [31]–[33]. As such, fast ice alone cannot often be adequately resolved by the coarse-resolution (25 km) satellite ice concentration data. For specific information on recent East Antarctic fast ice change and variability, please see [34]. That study presents a 2 km-resolution time series that covers a shorter period (2000–2008), and is based on 20-day compositing of satellite visible and thermal infrared imagery to remove cloud contamination.

## Results

### Mean Patterns of Seasonality

**Figure 1:**
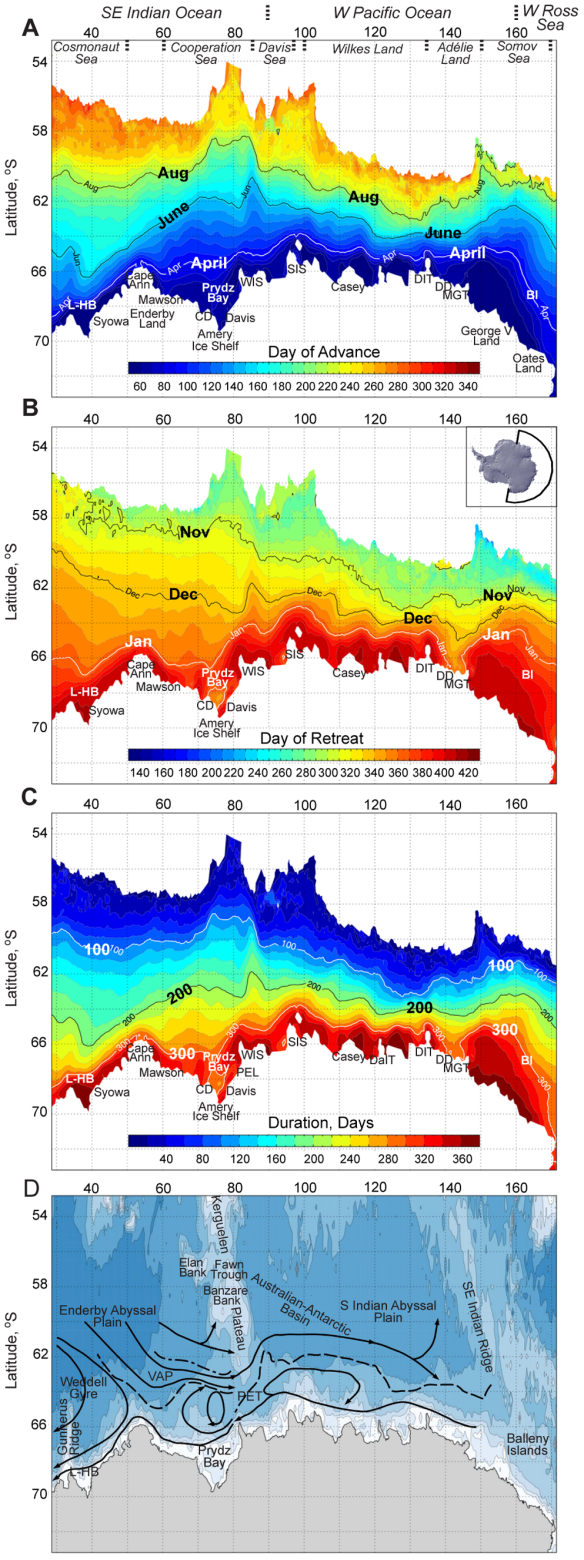
Climatological patterns of East Antarctic sea ice seasonality, 1979/80–2009/10. A) Mean days of sea ice advance, with contours for April, June and August marked. Place names used in the text are also marked: L-HB is Lützow-Holm Bay, CD Cape Darnley, PEL Princess Elizabeth Land, WIS West Ice Shelf, SIS Shackleton Ice Shelf, DaIT Dalton Iceberg Tongue, DIT Dibble Iceberg Tongue, DD Dumont d'Urville, MGT Mertz Glacier Tongue, and BI Balleny Islands). B) Mean days of sea ice retreat, with contours for November to January marked, and an inset of the study region. C) Mean ice season duration, with contours for 100, 200 and 300 days marked. D) Ocean bathymetry (contours at 500, 1000, 2000, 3000, 4000 and 5000 m), with a cartoon superimposed of the large-scale ocean circulation patterns in the sector determined from hydrographic measurements (after [28]). The dashed lines indicate the location of the SB-ACC, and the dash-dotted line that of the Southern Antarctic Circumpolar Current Front, VAP Valdivia Abyssal Plain, and PET Princess Elizabeth Trough. Numbers along the x axes are degrees longitude east.

To provide context (the background setting) for interpretation of observed change and variability presented in subsequent sections, we first present mean patterns of annual sea ice advance, retreat and duration for the period 1979/80–2009/10 in Figures 1A–C. This is presented within the context of previously-reported information on sea ice extent. As noted in previous studies (e.g., [35]) and shown in Figure 1, sea ice on average across East Antarctica is largely seasonal and generally forms a relatively narrow zone compared to other sectors at maximum extent – although the latter can vary substantially i.e., from∼54°S at∼80°E to∼61°S at∼135°E. This contrasts markedly with the Weddell and Ross seas, where major embayments extend to high latitudes and large (cyclonic) ocean gyres generate sea ice coverage that is up to∼20° of latitude in meridional extent [35]. In East Antarctica, the continental shelf is relatively narrow and the coastline relatively far north, ranging from∼70°S in Prydz Bay and Lützow-Holm Bay to∼66°S off Cape Ann (Figure 1D). Differences in the patterns of ice advance shown in Figure 1A result in sea ice cover that is on average three times more extensive at maximum extent at 80°E compared to 150°E (i.e., 18 versus 6 degrees of latitude), as noted previously in [36]. Relatively extensive coverage to the west∼100°E and east of∼150°E is separated by a zone that advances only about 500 km from the coast and is often only∼300 km wide in places. As described below, however, the East Antarctic sea ice zone may be narrow but it does not lack complexity in terms of patterns of seasonality in coverage.

The maps presented in Figure 1A–C reveal considerable regional variability in climatological patterns of annual advance, retreat and duration across East Antarctica. Major features of the ocean circulation setting (after [28]) are included in Figure 1D for comparison and to aid interpretation of the results, given the strong association noted in previous studies between sea ice distribution and ocean current patterns e.g., [28]. Major features of the oceanic circulation derived from the literature are depicted schematically on a map of bathymetry, to highlight the strong linkage between ocean currents and seafloor topography across this sector of the Southern Ocean (see [27]–[28], [37]–[39]).

As depicted in Figure 1D, regional oceanic circulation and thus patterns of sea ice drift (see [40]) are dominated by two circumpolar flow patterns, namely the westward-flowing Antarctic Coastal Current or East Wind Drift that skirts the continental margin to the south and the less constrained and eastward-flowing Antarctic Circumpolar Current (ACC) to the north. Flow throughout the study region is not purely zonal, however. The two major current systems are interconnected by a series of gyres and retroflections (e.g. gyres in the Prydz Bay region (∼75°E) and from∼85–115°E, and a northward deviation at∼85–90°E), and are separated by the Antarctic Divergence (AD). The position of the AD varies latitudinally but typically occurs at 63–65°S in the area of East Antarctica analysed here [41]. The Southern Boundary of the ACC (SB-ACC) occurs relatively close to the coast across the sector, but deviates north- and southwards within a window of approximately 4 degrees of latitude i.e., from∼66°S at 80°E to∼62°S at 90°E [27], [42].

Based on the spatial characteristics of sea ice seasonality in Figure 1, we identify three broad-scale regimes that display fairly distinctive and unique characteristics in terms of large-scale climatological patterns of sea ice advance, retreat and duration. These are: i) west of∼90°E; ii)∼90–145°E; and iii) east of∼145°E, although boundaries are somewhat indistinct. As discussed in more detail in the next section, mean patterns of seasonality within these sectors reflect their oceanic setting, and also tie in with those identified by Kimura and Wakatsuchi [43] regarding regional differences in processes contributing to the seasonal change in sea ice area around Antarctica. These are ice production/melt at the ice edge, ice production/melt within the sea ice zone, and zonal ice transport (lateral advection).

#### Mean Sea Ice Advance

As shown in previous studies (e.g., [35]), the distribution of ice in summer (the start of the annual sea ice season) is largely confined in East Antarctica to pockets on the continental shelf [35]. These are depicted in Figure 1C as those areas with durations of>360 days. Sea ice formation proper begins in March-April as air and ocean temperatures drop, and the ice edge advances to the north. At this time, the ice edge configuration is relatively smooth, with no significant regional deviations, and approximates the shape of the continental shelf break.

To the west of∼70°E, the mean pattern of sea ice advance is roughly from southeast to northwest for the first half of the year, after which time it becomes more zonal. Comparison of Figure 1A and 1D highlights the close association between large-scale patterns of climatological sea ice advance and ocean circulation and bathymetry that is a feature across all of East Antarctica (as previously noted by [28]). For example, note the association of the “kink” in early season ice advance (Figure 1A) with Gunnerus Ridge in the far south west of the region (Figure 1D). Along 70°E, sea ice advances by approximately 4° in latitude between the months of April and June, thereafter slowing to approximately half that speed. The cyclonic gyre off Prydz Bay is at least partly responsible for this pattern. The opposite pattern occurs along 40°E, with slow early advance and relatively rapid late advance, the latter probably due to eastward advection of sea ice into the region within the eastern limb of the Weddell Gyre (see [43]). As a result, sea ice is more extensive in autumn through winter in the east of the Southeast Indian Ocean sector (e.g. at 70°E) than the west (i.e. at 40°E), but the reverse is true by mid-winter. The relatively extensive mid-winter coverage west of∼50°E also mirrors a northward excursion of the Southern Boundary of the ACC (SB-ACC) there. By the same token, the sea ice attains its lowest latitude via a bulge centred on∼80°E that extends rapidly equatorwards from June through September. This appears to be related to the N–S trending Kerguelen Plateau and the associated Elan and Banzare banks, and an associated northward retroflection in the ACC (Figure 1D).

In contrast, mean advance in the region east of Kerguelen Plateau (from∼90 to∼145°E) is largely near-zonal early in the growth season, trending more from SE to NW as the season progresses. Rates of advance are proportionately similar to the SE Indian Ocean zone relative to the overall width of the sea ice zone at maximum extent, which is very narrow at these longitudes. The narrowness of the seasonal sea ice zone (zone of advance and retreat) in the region 115–145°E (Figure 1) is the result of a number of factors. These include a close correspondence in the locations of the SB-ACC and AD here [27], [44], and a southeastward veering of the ACC that brings warmer waters much closer to the coast to effectively constrain the coastal current to a relatively narrower band [28].

Climatological patterns of sea ice advance east of 145°E are distinctively different, but again mirror the geographical setting. Sea ice advance in this sector is multi-directional across a bulge centred on∼160°E. This appears to relate to strong topographic influence on ocean circulation, including a northward deflection of the ACC at∼140°E, and ice build-up of the SE Indian Ridge and Balleny Islands (Figure 1C). Moreover, the locations of the SB-ACC and the AD diverge substantially east of 140°E, as they do to the west of 85°E [27].

#### Mean Sea Ice Retreat

Although the relative rapidity of overall Antarctic sea ice retreat in late spring–summer is well known [45], there is again considerable variability in the pattern of mean retreat across the sector. This is illustrated in Figure 1B. The SE Indian Ocean sector is notable in terms of the rapidity of sea ice edge retreat. Along 40°E, for example, sea ice retreats on average by 10° of latitude in just two months (November through January), whereas it takes 4–5 months to advance along the same track (Figure 1A). In comparison, lower rates of retreat occurs on average in the largely narrower W. Pacific sea ice zone e.g., less than 2° of latitude along 130°E during the same three months (November through January), although sea ice advance over this track takes almost four months. In general, the pattern of retreat across the entire East Antarctic sector is in general relatively zonal as the season progresses, with the notable exception of the region to the west of∼50°E after mid-December where the trend is strongly from SE to NW. Later in the season, the mean pattern of retreat largely follows the trend of the continental shelf and Antarctic coastline. Relatively early seasonal sea ice retreat occurs in the vicinity of several coastal polynyas, notably in the Prydz Bay and Mertz Glacier regions.

#### Mean Sea Ice Duration

The mean patterns of ice season duration shown in Figure 1C reflect the combined processes of advance and retreat, with short duration periods in the outer pack ice and much longer periods close to the coast. However, given that advance occurs much more slowly than retreat, it is not surprising that the pattern of duration predominantly reflects that of advance rather than that of retreat. Exceptions to this do occur in several areas (see below). The broad band of outer pack ice that forms the marginal ice zone in winter (dark blues in Figure 1C) accounts for up to∼50% of the width of the sea ice zone in some places, e.g., at 130°E. However, this band of marginal ice zone persists for≤100 days only in a climatological sense. As we shall see in subsequent sections, the degree of duration varies substantially from year to year, and indeed from sector to sector across East Antarctica.

#### Correlation Analysis of Mean Advance, Retreat and Duration

**Figure 2:**
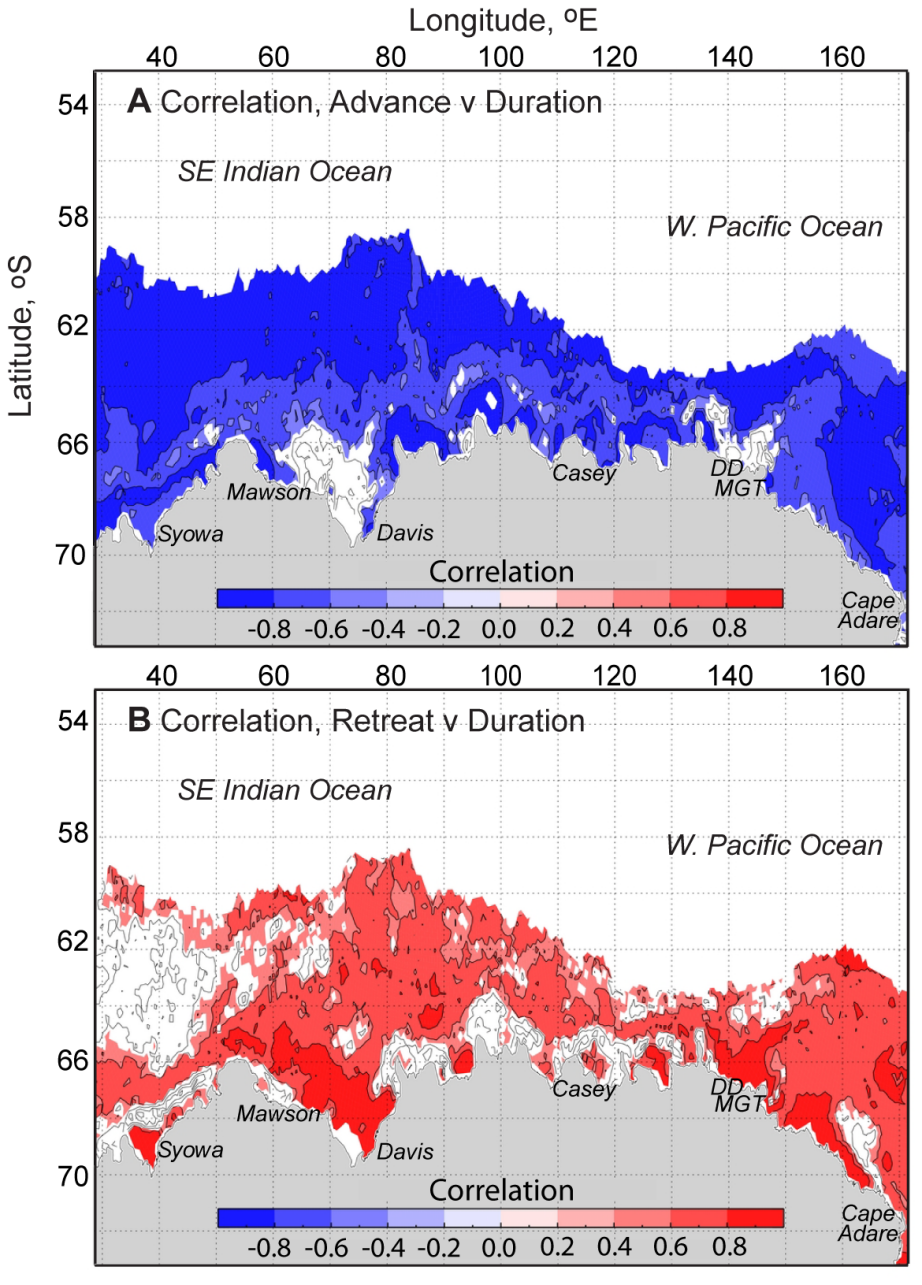
Colour-coded maps showing correlations of mean patterns of sea ice seasonality for the region 30–170°E and period 1979/80–2009/10. A) The correlation of mean annual duration versus day of sea ice advance. B) The correlation of mean annual duration versus day of sea ice retreat. Colour coding represents areas of statistical significance greater than the 99% level.

Correlation maps of mean (climatological) ice season duration versus annual advance and retreat for the 31-year time series are shown in Figure 2. These confirm that duration is in general more highly correlated to ice advance rather than retreat across most of East Antarctica. Specifically, there is high and statistically-significant negative correlation between advance and duration across the region (i.e., earlier advance largely relates to longer duration, and vice-versa), with the notable exception of areas in the western Prydz Bay-Mawson Coast, Shackleton Ice Shelf and Adélie Land-Mertz Glacier regions. Areas of lower correlation between advance and duration are regions of strong polynya activity.

Regions of strong polynya activity are also where correlations between retreat and duration (Figure 2B) are high and positive i.e., earlier retreat largely relates to shorter duration and vice-versa. Away from these coastal areas, correlations between retreat and duration are generally lower and more variable, or statistically insignificant. Particularly high (low) correlations between advance (retreat) and duration occur across an extensive region to the west of 50°E, an area influenced by the eastern margin of the Weddell Gyre (see Figure 1D and [40]). Here the retreat is particularly fast (Figure 1B) with low year-to-year variability, so it is the yearly variability in advance that co-varies more strongly with yearly variability in duration.

### Interannual Variability in Patterns of Seasonality

#### Anomaly Analysis

**Figure 3:**
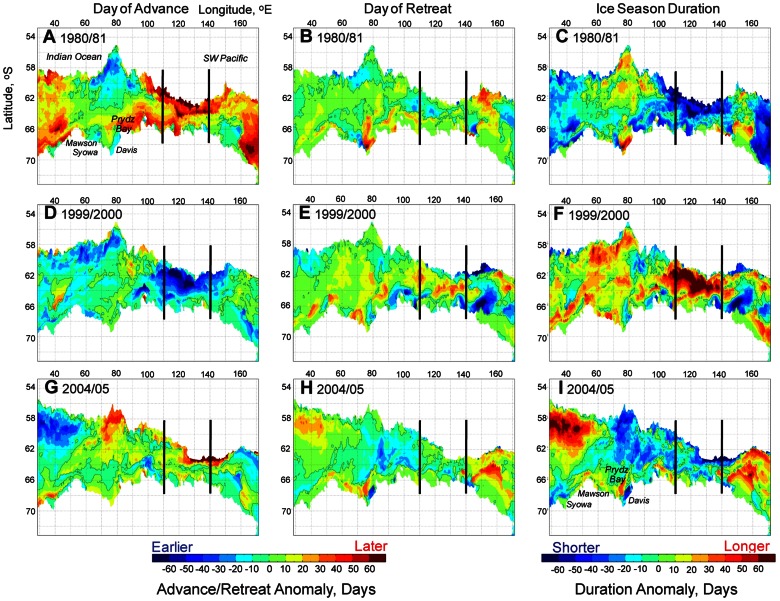
Year-to-year variability in East Antarctic sea ice seasonality shown in example maps of annual anomalies versus the long-term mean (1979/80–2009/10). A)–C) Anomaly maps of advance and retreat and resultant season duration, respectively, for 1980/81. D)–F) Anomaly maps of advance and retreat and resultant season duration, respectively, for 1999/2000. G)–I) Anomaly maps of advance and retreat and resultant season duration, respectively, 2004/05. The black lines depict the location of the meridional transects marked in Figure 4.

A fundamental feature of East Antarctic sea ice seasonality is large year-to-year variability. This is illustrated in Figure 3, with example anomaly maps from 1980/81, 1999/2000 and 2004/05. These years were chosen because they show particularly strong contrasting regional anomalies. Comparison of the ice season duration anomaly maps for 1980/81 (Figure 3C) and 1999/2000 (Figure 3F) reveals an important factor: although the two years are similar in terms of overall maximum ice extent, the patterns of duration across the region are strikingly different. For example, ice season duration in the relatively narrow band from∼100–145°E is as much as 60 or more days shorter than the long-term mean for that region in 1980/81, whereas it is up to>60 days longer in 1999/2000. This is a very large difference i.e.>4 months in terms of sea ice duration. In this case, the differing patterns of ice season duration in 1980/81 versus 1999/2000 are due in large part to anomalous patterns of sea ice advance (Figure 3A and D) rather than retreat; the latter are superficially similar for the two years (Figure 3B and E).

In Figure 3, a strong contrast is also observed in the large-scale patterns of ice season duration for 1980/81 versus 2004/05. In the latter year, two zones of strongly positive ice duration anomaly are present to the east of∼140°E and the west of∼60°E, separated by an extensive zone of negative anomaly (Figure 3I). While the negative anomaly remains a feature of ice season duration in 1980/81 in the region 100–150°E (Figure 3C), patterns elsewhere are quite different compared to 2004/05 (Figure 3I). A relatively localised “hot spot” of change appears to form off the Antarctic coast in the vicinity of Davis Station and between the Amery and West ice shelves (∼75°E–85°E); ice season duration anomalies there are strongly positive in 1980/81 and negative in 2004/05. In each of the three years shown, but particularly in 1980/81 and 2004/05, the duration anomaly in this location coincides with the location of the Barrier Polynya in southeastern Prydz Bay (off Davis Station; [46]) and is more related to variation in retreat than advance.

#### Interannual Variability at 110°E and 140°E

**Figure 4:**
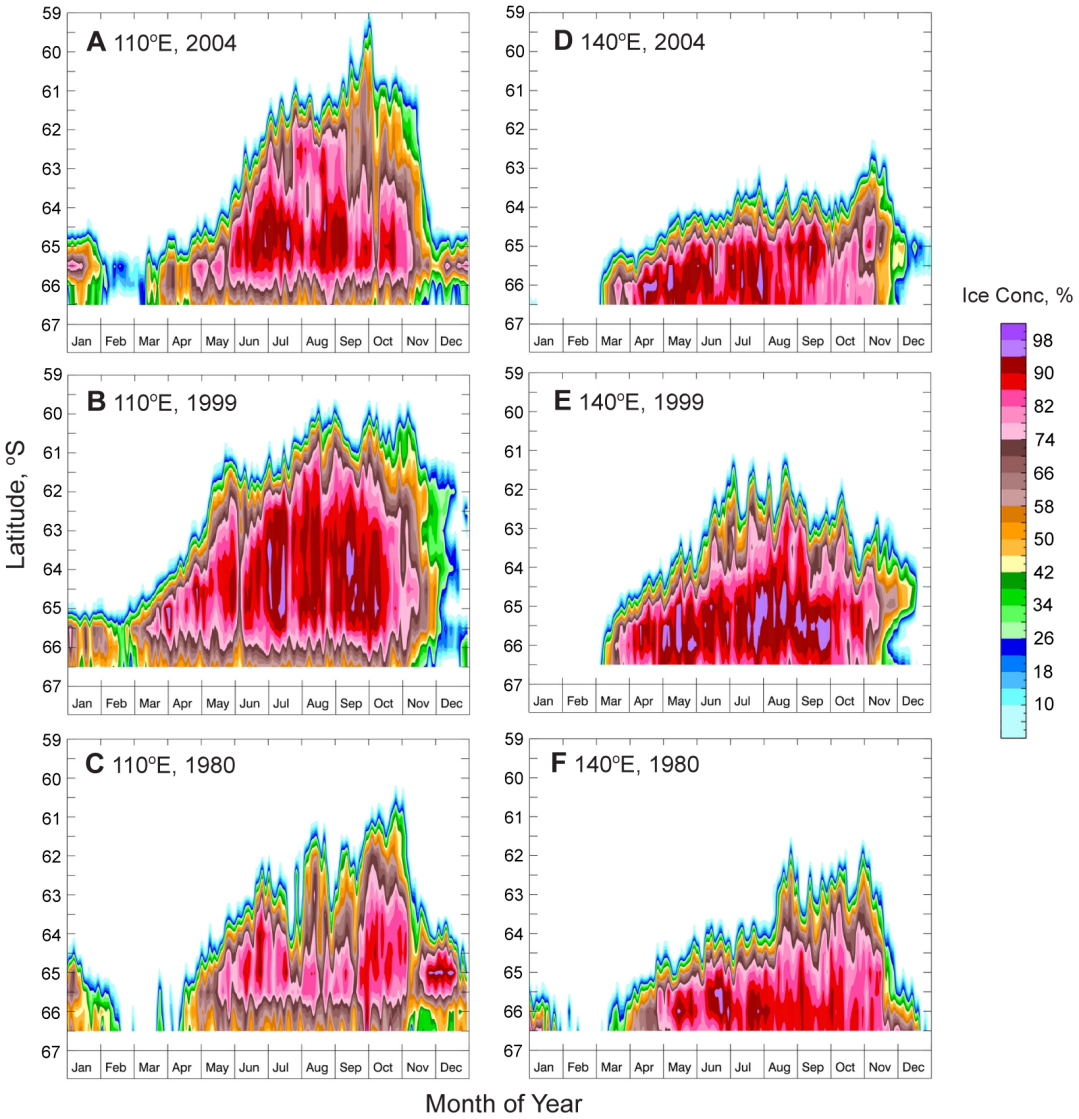
Examples of year-to-year variability in sea ice concentration and extent along the meridional transects shown in Figure 3. A)–C). Annual time series of daily ice concentration and extent “slices” along 110°E for 2004, 1999 and 1980, respectively. D)–F). Annual time series of daily ice concentration and extent “slices” along and along 140°E for 2004, 1999 and 1980, respectively.

The high degree of year-to-year variability is also reflected in Figure 4, which shows the same three annual periods, but here expressed as time series of daily ice concentration and extent “slices” along the 110°E and 140°E meridians from the ice edge to the continent. In 2004 and along 110°E (Figure 4A), a slow monotonic increase from a minimum extent in February–early March to a maximum in early October was followed by a rapid decline (particularly in November). In 1999, however, the pattern was quite different (Figure 4B); after a build-up phase from mid-February to mid-May, sea ice coverage remained at relatively low latitudes until a very rapid retreat occurred in December. Also apparent is substantial variability in the timing of maximum sea ice extent from year to year e.g. at 110°E: 1980 (∼60°S in late October), 1999 (59.2°S in late August), and 2004 (59°S in early October).

Large seasonal differences between years are also apparent along the 140°E meridian, which depicts a significantly different ice regime (Figure 4, right). In the three examples given, rapid ice advance consistently occurred from early–late March, but subsequent patterns of duration vary substantially from year to year in this narrow sea ice zone. Particularly striking is a relative plateauing of the coverage at 140°E in 2004 from early March until mid-November but particularly until early July (Figure 4D). In all cases, frequent synoptic-scale episodes of rapid advance and retreat of the ice edge by 1–2 degrees of latitude are consistent with the passage of storms (see [17]). The timing of maximum extent along 140°E is again highly variable, ranging from late August in 1980 and 1999 to early October in 2004.

#### Standard Deviations of Sea Ice Seasonality

**Figure 5:**
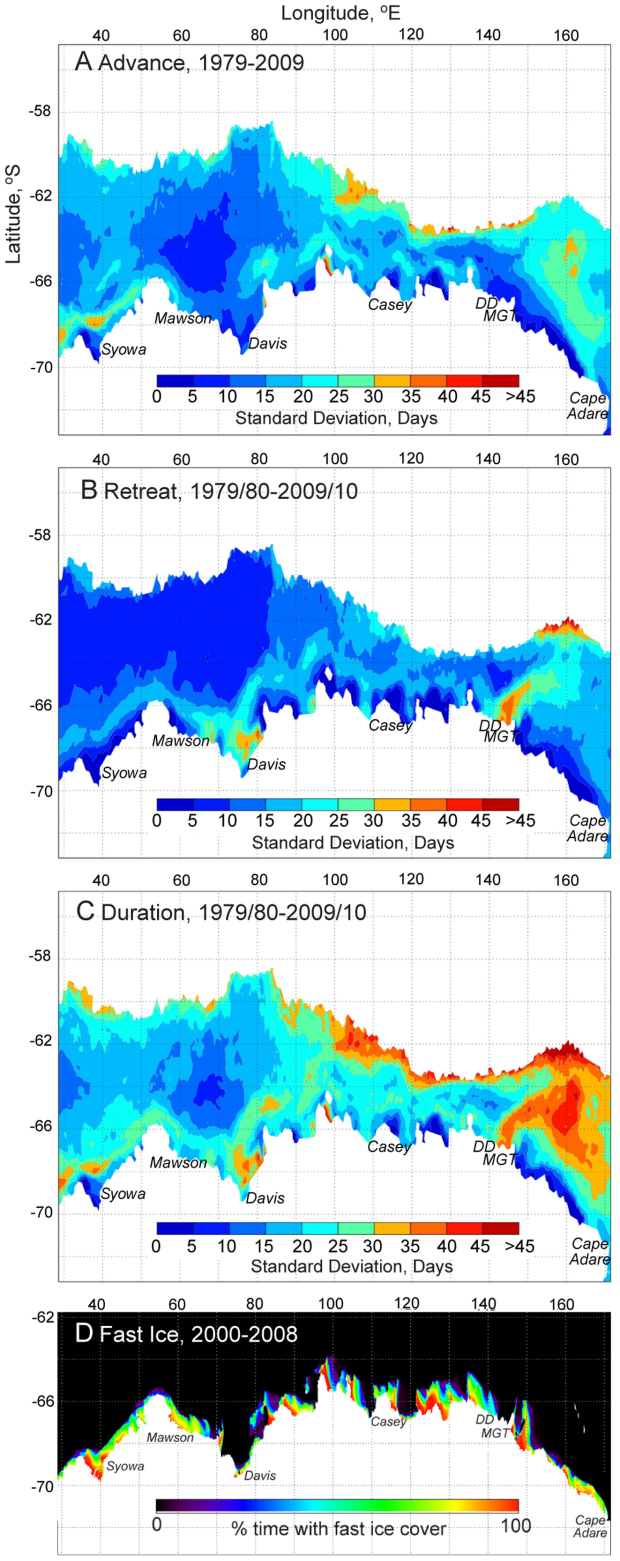
Variability in patterns of East Antarctic sea ice seasonality for the period 1979/80–2009/10. A)–C). Maps of standard deviation in days of annual sea ice advance, retreat, and season duration, respectively. D) Satellite-derived map of fast ice coverage averaged over the period March 2000 to December 2008, where a value of 100% is given to fast ice that covers the pixel for the entire 8.8 year period (after [34]).

Variability was investigated by mapping standard deviations (in days) of annual days of advance and retreat and ice season duration, and results are shown in Figure 5A–C. Values are low to moderately low across extensive regions of each of the maps (notably much of the offshore region west of∼90°E), and in retreat in particular. In the coastal zone, there is close correspondence between areas of low variability and fast ice distribution, the latter derived from the work of Fraser et al. [34] and presented for comparison in Figure 5D. There are also, however, marked “hot spots” of relatively high variability e.g., i) along the coast to the west of 50°E, in the marginal ice zones west of 75°E and between∼85°E and 150°E, and across the broad meridional band east of∼150°E in the advance map (Figure 5A); and in the Cape Darnley-Prydz Bay, Shackleton Ice Shelf and Mertz Glacier Polynya regions and the outer pack in the area 150–170°E in the retreat map (Figure 5B). These translate to considerable zonally-broad though latitudinally-narrow zones and local to regional-scale “hot spots” of high variability in ice season duration (Figure 5C). Of note is the strong contrast in variability in ice season duration between i) the sector to the west of∼85°E (low to moderate apart from the coastal and ice edge regions); ii) that from∼85–145°E (wherein high variability is largely confined to the marginal ice zone across a wide swath); and iii) the region east of∼145°E, which is almost exclusively high variability apart from the coastal strip that corresponds to fast ice shown in Figure 5D.

### Trends in East Antarctic Sea Ice Seasonality

**Figure 6:**
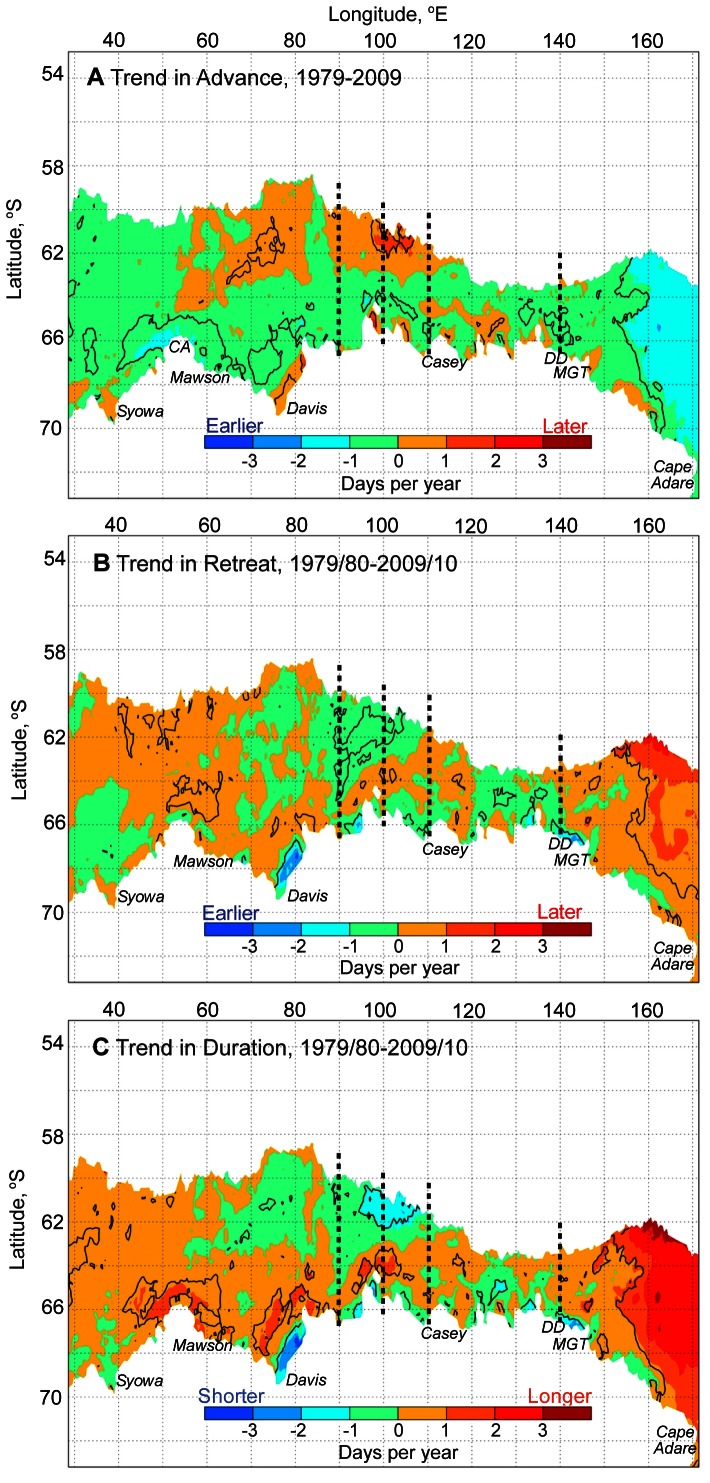
Trends in East Antarctic sea ice seasonality for the period 1979/80–2009/10. A)–C). Maps of trends in annual timings of East Antarctic sea ice advance, retreat, and duration, respectively. Contours denote statistical significance at the 95% level.

Maps of trends in annual timings of East Antarctic sea ice advance, retreat, and duration are shown in Figure 6. These results are based on the assumption of linearity over the period 1979/80 to 2009/10. In terms of advance and retreat, the region is characterised by mixed signals across an extensive zone, but with localised “hot spots” and strong regional contrasts. For example, there is a shortening of ice duration by 2–3 days per year off Davis Station between the Amery and West ice shelves, neighbouring a lengthening of the ice season by 2–3 days per year off the Cape Darnley region and in the coastal band to the west. A standout feature overall is the large zone of increasing sea ice season duration to the east of∼150°E; here, the trend is largely 2–3 days per year and greater. This corresponds to the western margin of the western Ross Sea sector highlighted by [6] and [18], and is consistent with their results. Moreover, the broad band of orange that dominates the region west of∼60°E and extends throughout much of the remaining sea ice zone signifies extensive moderate lengthening of the ice season duration, by≤1 day per year (Figure 6C).

In the outer half of the pack between∼60°E and 110°E, a general pattern of later advance and earlier retreat results in a general overall shortening of the ice season by 1 to 2 days per year, but this trend only reaches significance in the area between∼95–110°E. Along the coast there are other localized areas of change as well (in addition to the “hot spots” mentioned above). Ice season shortening is apparent between∼90°E and 150°E: to the east and west of the Mertz Glacier tongue (∼148°E), to the west of the Dibble Iceberg Tongue (∼135°E), in a corridor from 120–130°E), and adjacent to parts of the Shackleton Ice Shelf (centred on∼100°E). These areas correspond to locations of recurrent coastal polynyas [47]. In contrast, an increasing trend in duration of 1–2 days per year is apparent in the near-coastal zone from∼45°E to 60°E, from∼70°E to 88°E, and to the north of the Shackleton Ice Shelf (Figure 6C).

**Figure 7:**
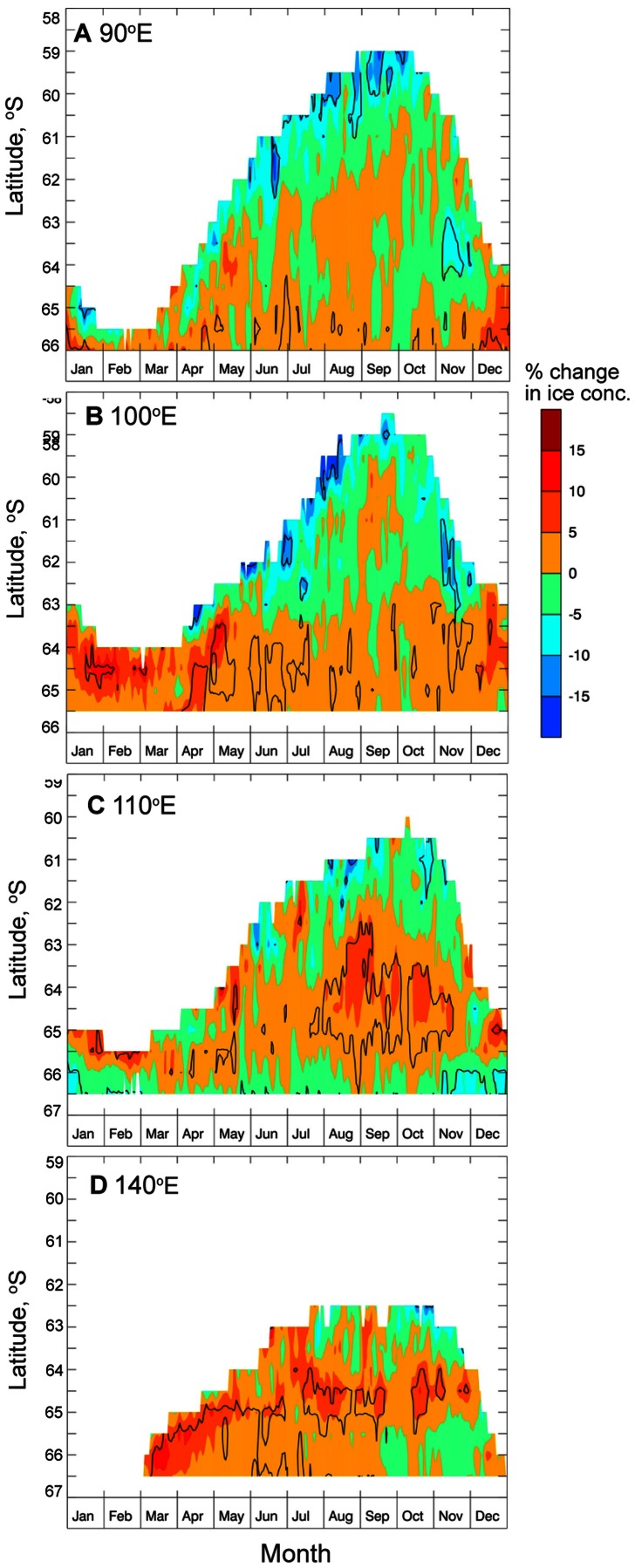
Trends in daily sea ice concentration shown along four meridional transects, for the period 1979–2010. A)–D). Trends in daily sea ice concentration along 90°E, 100°E, 110°E and 140°E, respectively. Contours denote statistical significance at the 95% level.

Further insight into the regional variability in observed trends in East Antarctic sea ice seasonality comes from analysis of coincident trends in daily ice concentration along four meridional transects that cut across different regimes as indicated in the trend maps shown in Figure 6 i.e., 90°E, 100°E, 110°E and 140°E (Figure 7). The 110°E and 140°E transects bisect regions of predominantly earlier ice advance, later retreat and increasing duration (Figure 6), and are characterised by positive ice concentration trends across fairly extensive zones that are largely located mid-pack, with negative trends in the outer pack and coastal margins (Figure 7C–D).

Along the 110°E transect, there is a contrasting decrease in sea ice concentration in the sea ice edge zone during winter maximum, albeit rather patchy (Figure 7C). This is again coincident with patterns shown in Figure 6 i.e., a trend towards a later sea ice advance in the outer pack, a later/longer winter maximum (September–October), and a later spring sea ice retreat (at both 110°E and 140°E), albeit largely non-statistically significant. The latter is perhaps assisted by an increase in more divergent winds at these latitudes and during the retreat (e.g., [48]), which would tend to delay ice edge retreat while opening the pack ice to the south (i.e., decreasing sea ice concentrations along the coast). Also evident, particularly along 110°E, is a trend for a decrease in sea ice concentration towards the coast from November through mid-January, again consistent with the tendency towards earlier spring retreat at those higher latitudes.

The pattern of trends along 100°E (Figure 7B) shows increased sea ice concentration through the southern part of the pack and a decrease in sea ice concentration in the outer pack during the advance and early retreat phases. In this case, however, a greater proportion of the outer pack is affected by the decreasing trend from April through November, apart from in September. This may reflect the fact that this transect bisects the “hot spots” in both negative and positive season duration trends, in the outer and mid to inner parts of the pack respectively (Figure 6C). Along another transect, at 90°E (Figure 7A), there is a slight decrease in sea ice concentration through much of the pack, while significant increases (decreases) are observed in the near-coastal zone (the marginal ice zone), similar to sea ice concentration trends at 100°E.

**Figure 8:**
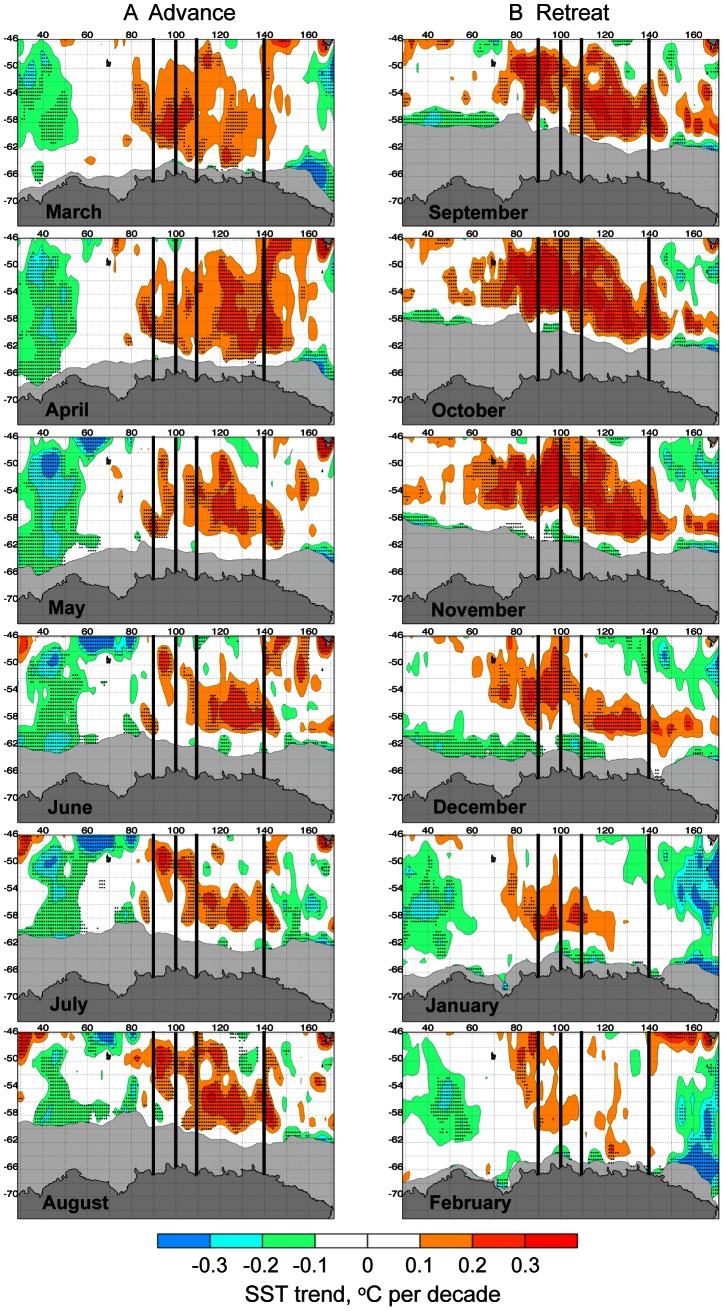
Monthly sea surface temperature (SST) trends north of the sea ice zone (marked in grey) for the period 1982–2010. A). Maps of SST trends for approximate months of annual sea ice advance (March–August). B). Maps of SST trends for approximate months of annual sea ice retreat (September–February). The x axis is degrees longitude, and the y axis degrees latitude. Hashed areas denote statistical significance at the 95% level. The black lines depict the location of the transects marked in Figure 7.

Although attribution is beyond the scope of this paper, we next carried out an initial analysis of possible relationships between trends in sea ice seasonality and sea surface temperatures (SSTs). Monthly SST trends (1982–2010) equatorward of the sea ice zone are shown in Figure 8 (and arranged as A period of sea ice annual “advance”, and B period of “retreat”), for comparison with Figures 6 and 7. Notable are trends towards slightly cooler SSTs just north of the ice edge at around 30–60°E from April through July, corresponding to earlier advance. Through much of the year, the sea ice edge in the western Ross Sea is also flanked by trends towards cooler SSTs, consistent with earlier advance and later retreat of sea ice in this area (Figure 6). A general pattern of SST warming is seen to the north of the ice edge from approximately 85–140°E during particularly during the winter maximum (August–October), corresponding to the late advance and early retreat in the outer pack ice at this location.

## Discussion

### Climatological Patterns of Sea Ice Seasonality in East Antarctica

Broadly speaking, mean patterns in sea ice seasonality off East Antarctica can be split into three different regimes: west of∼90°E,∼90–145°E, and east of∼145°E, although boundaries are somewhat indistinct. Within each regime, net patterns of seasonality are strongly related to patterns of oceanic circulation, which are in turn linked to bathymetry. These regional differences in seasonality in fact correspond to satellite-derived patterns of mean sea ice drift and ocean currents presented in [40]. For example, northward ice advance in prominent equatorward bulges in the vicinity of 80°E and east of 150°E in Figure 1 correspond to strong northward retroflections in surface ocean currents and associated sea ice drift (see also [49]–[51]). Our observations are also consistent with [43], who related seasonal sea ice changes to ice production/melt at the ice edge, ice production/melt within the sea ice zone, and zonal ice transport (lateral advection). In the following paragraphs, we assess mean patterns of sea ice advance, retreat and duration (Figure 1) in terms of the findings of [43].

In the SE Indian Ocean sector (30–90°E), rapid and extensive annual sea ice advance (Figure 1A) is largely driven by net sea ice production at the ice edge over a 4–5 month period (from March–April to July–August), with particularly strong production in the 30–60°E sector from June through August [43]. This rapid northward advance is supplemented by net westward transport of sea ice across the 60°E meridian for the months May through July, driven by easterly winds. The rapid areal expansion and subsequent maintenance of ice at lower latitudes (i.e., near maximum ice extent) to the east of∼50°E is also influenced by lateral advection of ice, but from west (the Weddell Sea), with eastward zonal transport across 60°E reaching a peak in October [40], [43]. In the SE Indian Ocean sector, the ice production phase is followed by net ice melt at the ice edge from September onwards but peaking on November–January. This is also consistent with the rapid rate of ice edge retreat that occurs over this period (Figure 1B). The zonal influx of ice from the Weddell Sea could contribute to the observed change in the direction of mean ice edge retreat at (and west of)∼50°E from zonal to NE to SW from December onwards, as observed in Figure 1B. More locally, polynyas in the Prydz Bay region are sites of intense ice formation during the advance phase, switching to intense melt during retreat.

Moving east, seasonal change in the relatively narrow sea ice zone from 90–150°E) reflects not only the patterns of ocean currents linked to bathymetry, but also the delicate, seasonally-varying balance between sea ice dynamics and thermodynamics. Mean patterns of ice edge advance observed across this sector in Figure 1A are largely determined by the production in coastal polynyas and in leads within pack ice that is subsequently advected offshore [43]. Net ice production at the ice edge here is confined to a short period in autumn and makes a small contribution to seasonal change in sea ice area compared to other Antarctic sectors, while ice melt occurs at the ice edge even during the advance season [43]. Zonal inflow of ice from adjacent regions is apparently a relatively minor component in terms of its effect on ice area change in the sector 90–145°E [43]. An increasing trend in ice edge melt throughout much of the season in the 90–145°E sector is largely consistent with the patterns of positive trend in SST off the ice edge shown in Figure 8, with an apparently strong relationship between the increase in SSTs and patterns of change in sea ice seasonality between 85°E and 110°E in particular. During spring-summer, sea ice retreat occurs rapidly as leads and coastal polynyas switch from sea ice “factories” [46] to focal points of enhanced ice melt [52] i.e. ice supply from the south diminishes and finally ceases. In this way, the Mertz Glacier polynya plays a major role in ensuring that sea ice retreats first to the coast in that region (see Figure 1B).

Finally and in the sector east of 145°E, the pattern of ice edge advance shown in Figure 1A is relatively rapid and occurs earlier in the year compared to the adjacent W Pacific Ocean sector. This again ties in with [43] i.e., strong ice production occurs at the ice edge over a 2-month period in early autumn only (March–April) in this sector. Patterns of seasonality in this complex eastern part of the study region are influenced not only by unique characteristics of the geographical setting (patterns of ocean currents and seafloor bathymetry and the extensive build-up and presence of perennial sea ice) but also by westward influx of ice into the region from the Ross Sea [43], [53]. This zonal influx is most prominent in the November to April period - that is, through much of the period when annual ice production has largely ceased, to help supplement/maintain the perennial presence of ice there. Other key physical factors affecting mean patterns of sea ice seasonality here are a northward retroflection in the SB-ACC east of∼140°E, the effect on ocean currents of a widening of the continental shelf and the offshore presence of the extensive SE Indian Ridge system, a dominant northward retroflection of mean ocean surface currents and associated ice drift in the region 145–160°E, 61–65°S [40], and the presence of the Balleny Islands.

### Change and Variability in East Antarctic Sea Ice Seasonality

As shown in this study, the pattern of change in sea ice seasonality across the East Antarctic sector is considerably more complex than the well-documented trends from West Antarctica shown by [5]–[6] and [18]. These are centred on relatively large and more homogeneous regions of change in the Antarctic Peninsula-Bellingshausen Sea (shortening ice season duration by∼3 days per year since 1979/80) and western Ross Sea (lengthening duration by∼3 days per year). The latter encroaches on the eastern part of our study area, and represents a strong contrast in terms of its size and relative homogeneity compared to the East Antarctic sea ice zone, where the pattern of change in sea ice seasonality comprises mixed signals which are regionally to locally significant.

In East Antarctica, regions of significant shortening of the sea ice season, by 1 to 3 days per year over the 31-year period, are limited to relatively small pockets along the ice sheet coastal margin between∼75°E and 150°E and a more extensive sector of the outer pack offshore from Wilkes Land (between 95°E and 110°E) (Figure 6C). The latter sits within a larger region of non statistically-significant weaker trends (shortening by up to 1 day per year) that includes the outer Prydz Bay regime (over the Kerguelen Plateau). The negative trends in duration are driven by trends towards both later annual advance and earlier retreat. The coastal “hot spots” include the Princess Elizabeth Land coast between the Amery and West Ice Shelves (in the vicinity of Davis Station), the Adélie Land coast (135–150°E) and areas adjacent to the Shackleton Ice Shelf.

Although geographically limited, “hot spots” of negative trends in sea ice seasonality along the coast observed between∼75–150°E are of considerable interest for a number of reasons. For example, the near-coastal “hot spots” appear to coincide with the location of certain important coastal polynyas e.g., the Barrier Polynya in eastern Prydz Bay and the Mertz Glacier Polynya centred on∼145°E. Such polynyas are important as sites of enhanced sea ice production [46], seasonal melt (Figure 1B and [52]) and biological productivity [54]. The Mertz Glacier Polynya is also globally important as a key producer of Antarctic Bottom Water [55]–[56]. Moreover, important penguin breeding sites are associated with polynyas [47]. In the case of the Barrier Polynya, change in local sea ice production rates and associated water mass modification have been shown to have an important effect on reducing incursions of warm Circumpolar Deep Water to the underside of the Amery Ice Shelf [57]. Intriguingly, no significant trend is apparent in the wintertime size of the Barrier Polynya for the period 1992–2008 [58].

By contrast, areas of positive trend in sea ice duration are more extensive in East Antarctica than areas of negative duration for the overall (31-year) period, and appear to be associated with both earlier advance and later retreat. In addition to the extensive area of lengthening in the Western Ross Sea regime, other key regions of ice season lengthening occur in the near-coastal zone to the west of∼105°E and particularly between∼40°E and 90°E, in the vicinity of Cape Ann. This change may relate to change in behaviour of the Cosmonaut Polynya, an offshore sensible heat polynya that has an average location centred on 56°E and 65°S [59]–[60]. An intriguing finding is the near juxtaposition in certain regions of the near-coastal zone from∼40°E to 110°E of pockets of strongly positive and negative trends in sea ice duration e.g., in Prydz Bay and off the Wilkes Land coast (Figure 6C). The strongest west/east contrast is within Prydz Bay, where (to the west) Cape Darnley polynya shows increasing ice season duration, while (to the east) the Barrier Polynya (as noted above) shows decreasing ice season duration. The Cape Darnley polynya has recently been described as an area of significant bottom water production [61]. The strong positive trend in ice season duration, particularly just to the northeast of Cape Darnley polynya, is consistent with the observations in [61] of intense sea ice production within the polynya.

In terms of annual sea ice advance and retreat, there is a predominant trend towards earlier sea ice advance through much of the central pack ice region from 30–150°E, although this is statistically significant in only a few localized pockets (Figure 6A–B). The outer part of the sea ice zone from 55–120°E shows a trend for later advance, although again significant only in a few small areas. For retreat, the situation is somewhat reversed, with a trend for earlier retreat in some parts of the outer ice edge (notably 90–110°E and 120–135°E) and a trend towards later retreat outside these regions.

Some insight into the regional variability in observed trends in East sea ice seasonality in the W Pacific sector comes from statistically-significant trends towards increasing mid-pack sea ice concentration along 110°E and 140°E shown in Figure 7C–D, and their comparison with Figure 6. Given that intra-pack melting within leads is an important seasonal driver of Antarctic sea ice retreat [62], we speculate that such a decrease in open water over much of the central pack (and late in the season in particular) may inhibit sea ice retreat. For example, increased mid-pack concentration (possibly due to increased ice convergence or lateral input of ice) reduces the amount of open water present, which in turn reduces melt within leads, delaying retreat [63]. In addition, the difference in patterns of sea ice concentration change along four transects (90°E, 100°E, 110°E and 140°E shown in Figure 7) again mirrors the complexity and range of variability in change in sea ice seasonality in East Antarctica (Figure 6), in that they are from the same regime (∼90–145°E).

The broader trend towards later sea ice advance and earlier sea ice retreat in the outer pack is consistent with an observed trend for increased SSTs to the north of the ice edge from August to October, which would inhibit maximum winter ice edge extent and initiate early retreat (melt back). This area of late advance was also recently highlighted in a study describing wind-driven trends in Antarctic sea ice drift [64]. That study showed winds, ice motion and ice concentration to be aligned in most locations around Antarctica (e.g., wind-driven northward ice flow showing increased sea ice concentration) except in the sector∼90°E–120°E, where mean northerly winds appear to oppose mean northward ice motion. However, the strong westward flowing coastal current likely explains the mean northward ice motion in this location, while the northerly winds and warm SSTs help to explain the late advance, early retreat and short ice season. Thus, our observations lend support and insight regarding why the 90°E–120°E sector is exceptionally anomalous as highlighted by [64].

Where there is shown to be a decrease in SSTs to the north of the ice edge (at around 40°E for example), there is an earlier advance and a later retreat (Figures 6A–B and 8). The trends towards warmer SST and later/earlier advance/retreat do not fully overlap, however. Given that that SST trends may be contributing to changes in sea ice seasonality along the East Antarctic coast, we are unsure if the reported dominance of ice edge reduction over production in certain sectors [43] can be extrapolated beyond the period of their study (2003–9). As noted earlier, sea ice advance, retreat and concentration are also influenced by dynamic processes relative to wind direction; investigation of this is the focus of the planned next stage of this work.

### The Role of “Icescape Elements”

The apparently close relationship between “hotspots” of changing sea ice seasonality and polynyas noted above points to the importance of accounting for change and variability in the different elements comprising the sea ice zone, and their interaction. This steps beyond simply treating the sea ice zone as an amorphous mass that is delineated by an ice edge. These “icescape elements” include the outer pack or marginal ice zone, the inner pack and the near-coastal zone including areas of fast ice and polynyas (both coastal latent heat and offshore sensible heat) [17], [65].

Complex patterns of change and variability in sea ice seasonality are observed on the continental shelf and within the Antarctic Coastal Current. Here, sea ice distribution and characteristics are strongly affected by interaction with the ice sheet margin and icebergs [66]. Generally speaking, dynamic build-up of thick consolidated sea ice (particularly fast ice [34]) tends to occur to the east of coastal promontories i.e., upstream, while coastal polynyas form to the west [67]. Moreover, groups of icebergs grounded in waters shallower than∼400 m strongly affect both polynya and fast ice distribution, and as far offshore as 200–300 km in places [32], [66], [68].

Within this coastal zone, the largest variability in days of retreat and ice season duration coincides with polynya locations. The Mertz Glacier polynya regime, for example, has a dominant effect on regional sea ice retreat and its variability around 140–155°E (Figures 5B–C and 6B–C; see also [52]). As shown in [52], strong interannual variability in the size and configuration of this polynya relates to wind strength and direction as it affects the polynya and surrounding regional “icescape” (see also [69]). The major “hot spot” in sea ice duration variability (and shortening) off the Princess Elizabeth Land coast between the Amery and West Ice Shelves and in the vicinity of Davis Station (Figures 5C and 6C) also appears to be partly associated with polynya activity there, again possibly linked to grounded icebergs. An additional factor is that change in one “icescape element” can have a major impact on other elements and the regional icescape in general. As a case in point, the large-scale calving of the Mertz Glacier Tongue in early 2010 had a major and abrupt effect on sea ice conditions in the region [70], with longer-term consequences yet to be realized.

In contrast to polynyas, areas of low sea ice variability in the coastal zone correspond to regions of fast ice, particularly during the retreat phase (Figure 5B). Information on change and variability in the areal distribution of East Antarctic fast ice is given in [34]. This dataset covers the period March 2000–December 2008 and comprises a time series of 20-day composite maps that reveal a number of interesting patterns in fast ice coverage across the region. Overall fast ice extent showed a slight increase of 1.4% per year over the 9-year period, with a stronger increasing trend in the SE Indian Ocean sector (20–90°E, 4.1% per year) compared to the W Pacific sector (90–160°E). Interannual variability is generally lower in the SE Indian Ocean sector, but increased after 2004; this change coincided with an increase in fast ice persistence (survival) through summer after that time. In contrast, interannual variability is much higher throughout the 9-year time series in the W Pacific sector. This study underscores the need for continued monitoring of fast ice changes.

In general, regions of thick consolidated sea ice occurring in regions of net convergence (including dynamically-formed fast ice) or fast ice in more sheltered locations have a higher likelihood of surviving longer than regions of predominantly diffuse and thinner ice e.g. within the marginal ice zone. This is illustrated by the occurrence off the George V and Oates Land coasts of the largest area of multi-year sea ice in East Antarctica (Figure 1; see also [35]). Persistence of this body of thick, highly-deformed and –consolidated ice is also affected by the presence of the Balleny Islands. By contrast, high variability in sea ice duration is observed in wide swaths of the marginal ice zone across East Antarctica (Figure 5C), where ocean waves act as a dominant agent of both sea ice formation and destruction [71]. Marginal ice zone configuration and extent is also strongly affected on the synoptic scale by the passage of storms, as shown in Figure 3.

**Figure 9:**
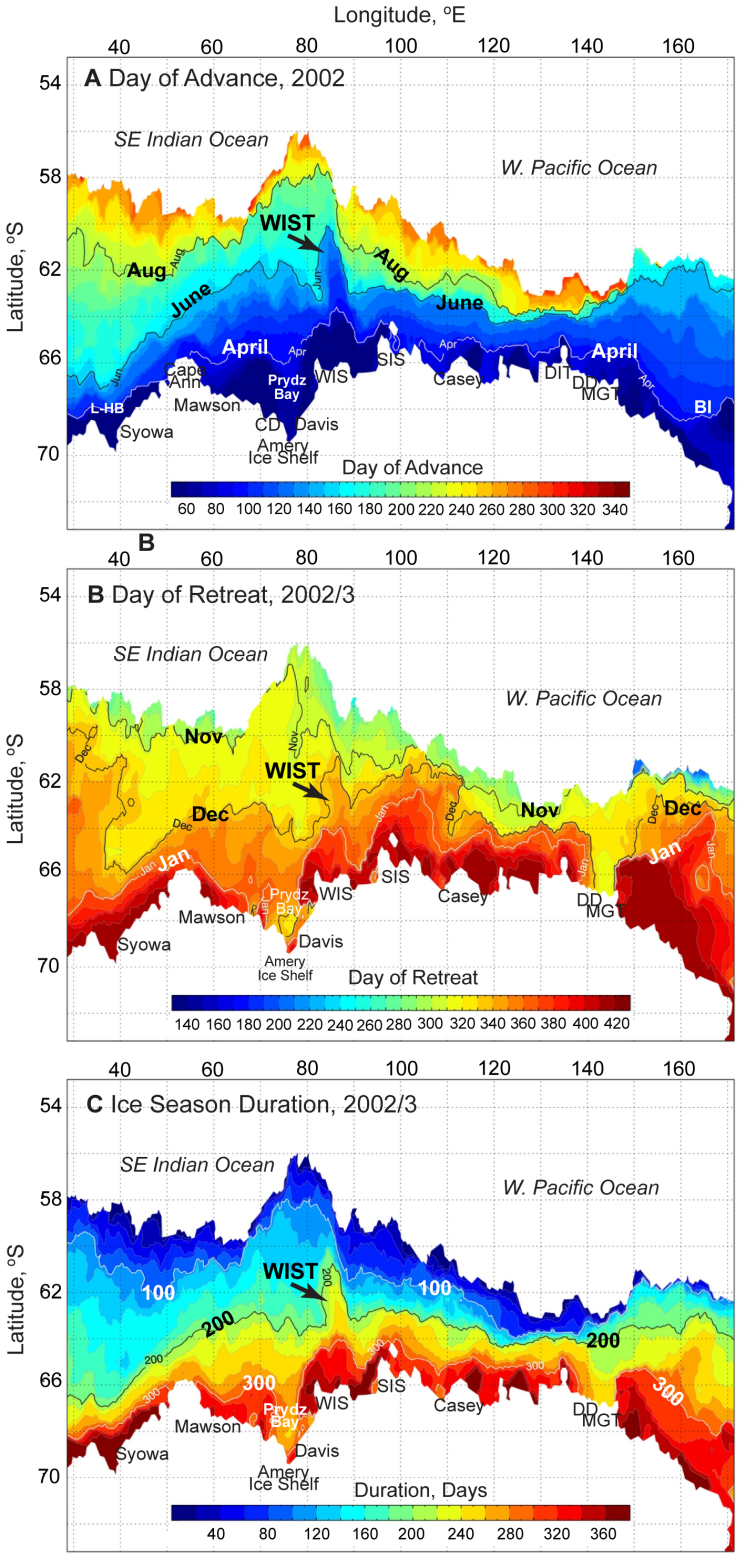
Maps showing the dominant regional effect on sea ice seasonality of the West Ice Shelf Tongue (WIST). A). Days of sea ice advance for 2002, with April, June and August contours marked. B). Days of sea ice retreat for 2002/03, with November, December and January contours marked. C). Sea ice season duration for 2002/03, with the 100-, 200- and 300-day contours marked.

Another important offshore element is the so-called West Ice Shelf Tongue (WIST), a prominent meridionally-trending sea ice tongue which recurs and persists annually in the vicinity of 85°E [72]. The dominant regional impact of the WIST on sea ice advance, retreat and duration in this location is illustrated in maps from 2002 (Figure 9). It was particularly well represented in that year, when it developed over a period of 30 days in April–May to extend northwards from, and perpendicular to, the surrounding ice edge for more than 800 km and covered an area greater than 200,000 km^2^. Figure 9 shows that WIST advances substantially earlier than the surrounding pack ice and retreats later, resulting in substantially longer ice duration. The WIST forms as the result of sea ice advection in a sharp northward turning of the southern part of the ACC near 85°E, around the southeastern edge of the Kerguelen Plateau ([72]; also see Figure 1D). Year-to-year variability in the meridional extent of the tongue is largely caused by variations in the autumn and early winter winds, with southerly (northerly) anomalies resulting in large (poorly-developed) sea ice tongues [72].

The WIST illustrates the influence of complex interactions between large-scale ocean and atmosphere circulation patterns on the seasonal development and maintenance of the East Antarctic sea ice cover. Sea ice advance, retreat and concentration are affected not only by thermodynamic but also by dynamic processes [43]. Whereas offshore winds lead to ice divergence and transport colder air northwards across the ice to extend the pack, dominant on-ice winds not only lead to ice convergence (higher concentration and ice edge retreat) but also warmer conditions [73]–[74]. Clearly, more work is necessary to unravel factors driving the seasonal sea ice patterns and trends across the East Antarctic sector, including change and variability in large-scale atmospheric and oceanic circulation patterns and possible teleconnections with mid- to low-latitude processes.

## References

[1] Lubin D, Massom RA (2006) Polar remote sensing Volume I: Atmosphere and oceans. Chichester and Berlin: Praxis/Springer-Verlag. 756 p.

[2] Thomas DN, Dieckmann GS, editors (2010) Sea Ice, 2^nd^ Edition. Oxford: Wiley-Blackwell. 621 p.

[3] Comiso JC (2010) Variability and trends of the global sea ice cover. In: Thomas DN, Dieckmann GS, editors. Sea Ice, Second Edition.Oxford: Wiley-Blackwell. pp. 205–246.

[4] Comiso JC, Kwok R, Martin S, Gordon AL (2011) Variability and trends in sea ice extent and ice production in the Ross Sea. J Geophys Res 116: C04021 doi:10.1029/2010JC006391

[5] Parkinson CL (2002) Trends in the length of the Southern Ocean sea-ice season, 1979–99. Ann Glaciol 34: 435–440.

[6] Stammerjohn S, Massom R, Rind D, Martinson D (2012) Regions of rapid sea ice change: An inter-hemispheric seasonal comparison. Geophys Res Lett 39: L06501 doi:10.1029/2012GL050874

[7] Perovich DK (2011) The changing Arctic sea ice cover. Oceanogr 24(3): 162–173 Available: http://dx.doi.org/10.5670/oceanog.2011.68.Accessed January 23, 2013.

[8] Maksym T, Stammerjohn SE, Ackley S, Massom RA (2012) Antarctic sea ice – A polar opposite? Oceanogr 25(3) 140–151 Available: http://dx.doi.org/10.5670/oceanog.2012.88.Accessed January 28, 2013.

[9] Perovich DK, Richter-Menge JA, Jones KF, Light B (2008) Sunlight, water and ice: Extreme Arctic sea ice melt during the summer of 2007. Geophys Res Lett 35: L11501 doi:10.1029/2008GL034007

[10] Montes-Hugo M, Doney SC, Ducklow HW, Fraser W, Martinson DG, et al (2009) Recent changes in phytoplankton communities associated with rapid regional climate change along the Western Antarctic Peninsula. Science 323: 1470–1473 doi: 10.1126/science.1164533 19286554 10.1126/science.1164533

[11] Meredith MP, King JC (2005) Rapid climate change in the ocean west of the Antarctic Peninsula during the second half of the 20th century. Geophys Res Lett 32(19) : DOI: 10.1029/2005GL024042

[12] Martinson DG (2011) The Antarctic Circumpolar Current's role in the Antarctic ice system: An overview. Paleog, Paleoclim, Paleoecol 335–336: 71–74.

[13] Rintoul SR, Sparrow M, Meredith MP, Wadley V, Speer K, et al.. (2012) The Southern Ocean Observing System: Initial science and implementation strategy. 74 p.

[14] Martinson DG, Stammerjohn SE, Iannuzzi RA, Smith RC, Vernet M (2008) Western Antarctic Peninsula physical oceanography and spatio-temporal variability. Deep-Sea Res II 55: : 1,964–1,987.

[15] Pritchard HD, Ligtenberg SRM, Fricker HA, Vaughan DG, van den Broeke MR, et al. (2012) Antarctic ice-sheet loss driven by basal melting of ice shelves. Nature 484: , 502–505. 10.1038/nature10968 22538614

[16] Dinniman MS, Klinck JM, Hofmann EE (2012) Sensitivity of Circumpolar Deep Water transport and ice shelf basal melt along the West Antarctic Peninsula to changes in winds. J Clim 25: 4799–4816.

[17] Massom RA, Stammerjohn S (2010) Antarctic sea ice change and variability – Physical and ecological implications. Polar Science 4(2): 149–186.

[18] Stammerjohn SE, Martinson DG, Smith RC, Rind D (2008a) Trends in Antarctic annual sea ice retreat and advance and their relation to ENSO and Southern Annular Mode variability. J Geophys Res 113: C03S90 doi: 10.1029/2007JC004269

[19] Stammerjohn SE, Martinson DG, Smith RC, Yuan X, Iannuzzi RA (2008b) Sea ice in the western Antarctic Peninsula region: Spatio-temporal variability from ecological and climate change perspectives. Deep-Sea Res II 55: 2041–2058.

[20] Ducklow H, Clarke A, Dickhut R, Doney SC, Geisz H, et al.. (2012) The marine ecosystem of the West Antarctic Peninsula. In: Rogers A, Johnston N, Clarke A, Murphy E, editors. Antarctica: An extreme environment in a changing world. London: Blackwell. pp. 121–159.

[21] McClintock J, Ducklow H, Fraser W (2008) Ecological impacts of climate change on the Antarctic Peninsula. Am Sci 96: 302–310.

[22] Stephens BB, Keeling RF (2000) The influence of Antarctic sea ice on glacial-interglacial CO_2_ variations. Nature 404: 171–174. 10724166 10.1038/35004556

[23] Delille B, Jourdain B, Borges AV, Tison J-L, Delille D (2007) Biogas (CO_2_, O_2_, dimethylsulfide) dynamics in Spring Antarctic fast ice. Limnol Oceanogr 52(4): 1367–1379.

[24] Arrigo KR, van Dijken GL, Long M (2008) Coastal Southern Ocean: A strong anthropogenic CO_2_ sink. Geophys Res Lett 35: L21602 doi:10.1029/2008GL035624

[25] Dieckmann GS, Nehrke G, Papadimitriou S, Göttlicher J, Steininger R, et al (2008) Calcium carbonate as ikaite crystals in Antarctic sea ice. Geophys Res Lett 35: L08501 doi:10.1029/2008GL033540

[26] Parkinson CL (1998) Length of the sea ice season in the Southern Ocean. In: Jeffries MO, editor. Antarctic sea ice physical processes, interactions and variability. Antarctic Research Series 74. Washington DC: American Geophysical Union. pp. 173–186.

[27] Bindoff NL, Rosenberg MA, Warner M J (2000a) On the circulation of the waters over the Antarctic continental slope and rise between 80 to 150°E. Deep-Sea Res Part II 47(12–13): 2299–2326.

[28] Nicol S, Raymond B (2012) Pelagic ecosystems in the waters off East Antarctica (30°E–150°E). In: Rogers AD, Johnston NM, Murphy EJ, Clarke A, editors. Antarctic ecosystems: An extreme environment in a changing world. Oxford: Wiley Blackwell. DOI: 10.1002/9781444347241.ch8

[29] Amante C, Eakins BW (2009) ETOPO1 1 arc-minute global relief model: Procedures, data sources and analysis. NOAA Technical Memorandum NESDIS NGDC-24, 19 pp, March 2009

[30] Reynolds RW, Smith TM (1994) Improved global sea surface temperature analyses using optimum interpolation. J Climate 7: 929–948.

[31] Ushio S (2006) Factors affecting fast-ice break-up frequency in Lützow-Holm Bay, Antarctica. Ann Glaciol 44: 177–182.

[32] Giles AB, Massom RA, Lytle VI (2008) Fast ice distribution in East Antarctica during 1997 and 1999 determined using Radarsat data. J Geophys Res 113: C02S14 doi:10.1029/2007JC004139

[33] Massom RA, Giles AB, Fricker HA, Warner RC, Legresy B, et al (2010) Examining the interaction between multi-year landfast sea ice and the Mertz Glacier Tongue, East Antarctica: Another factor in ice sheet stability? J Geophys Res 115: C12027 doi:10.1029/2009JC006083

[34] Fraser AD, Massom RA, Michael KJ, Galton-Fenzi BK, Lieser JL (2012) East Antarctic landfast sea ice distribution and variability, 2000–08. J. Climate 25: 1137–1156.

[35] Gloersen P, Campbell WJ, Cavalieri DJ, Comiso JC, Parkinson CL, et al.. (1992) Arctic and Antarctic sea ice, 1978–1987: Satellite passive-microwave observations and analysis. NASA Special Publication SP-511. Washington DC: NASA. 290 p.

[36] Nicol S, Pauly T, Bindoff NL, Wright S, Thiele D, et al (2000) Ocean circulation off East Antarctica affects ecosystem structure and sea-ice extent. Nature 406: 504–507. 10952309 10.1038/35020053

[37] McCartney MS, Donohue KA (2007) A deep cyclonic gyre in the Australian–Antarctic Basin,. Prog Oceanogr 75: 675–750 doi:10.1016/j.pocean.2007.02.008

[38] Meijers AJS, Klocker A, Bindoff NL, Williams GD, Marsland SJ (2010) The circulation and water masses of the Antarctic shelf and continental slope between 30 and 80°E. Deep-Sea Res II.57: : 9–10, 723–737.

[39] Williams GD, Nicol S, Bindoff NL, Aoki A, Meijers AJS, et al (2010) Surface oceanography of BROKE-West, along the Antarctic margin of the South-West Indian Ocean (30–80°E). Deep-Sea Res II 57: 738–757.

[40] Kimura N (2004) Sea ice motion in response to surface wind and ocean current in the Southern Ocean. J Met Soc Japan 82: 1223–1231.

[41] Allison I (1997) Physical processes determining the Antarctic sea ice environment. Austr J Phys 50: 759–771.

[42] Orsi AH, Whitworth III T, Nowlin WD Jr (1995) On the meridional extent and fronts of the Antarctic Circumpolar Current. Deep-Sea Res I 42: 641–673.

[43] Kimura N, Wakatsuchi M (2011) Large-scale processes governing the seasonal variability of Antarctic sea-ice area. Tellus 63A: 828–840.

[44] Nicol S, editor BROKE: A multidisciplinary survey of the waters off East Antarctica (80–150°E). Deep-Sea Res II 47(12–13): 2281–2613.

[45] Gordon AL (1981) Seasonality of Southern-Ocean sea ice. J Geophys Res 86(NC5): 4193–4197.

[46] Tamura T, Ohshima KI, Nihashi S (2008) Mapping of sea ice production for Antarctic coastal polynyas. Geophys. Res. Lett. 35: L07606 doi:10.1029/2007GL032903

[47] Massom RA, Harris PT, Michael K, Potter MJ (1998) The distribution and formative processes of latent heat polynyas in East Antarctica. Ann Glaciol 27: 420–426.

[48] Watkins AB, Simmonds I (1999) A late spring surge in the open water of the Antarctic sea ice pack. Geophys Res Lett 26: 1481–1484.

[49] Tchernia P, Jeannin PF (1984) Circulation in Antarctic waters as revealed by iceberg tracks 1972–1983. Polar Rec 22 (138): 263–269.

[50] Young NW, Hyland G (1997) Applications of time series of microwave backscatter over the Antarctic region. Eur. Space Agency Spec. Publ 414: 1007–1014.

[51] Heil P, Allison I (1999) The pattern and variability of Antarctic sea-ice drift in the Indian Ocean and western Pacific sectors. J Geophys Res.104(C7): : 15,789–15,802.

[52] Massom RA, Jacka K, Pook MJ, Fowler C, Adams N, et al (2003) An anomalous late-season change in the regional sea ice regime in the vicinity of the Mertz Glacier Polynya, East Antarctica. J Geophys Res 108(C7): 3212 doi:10.1029/2002JC001354

[53] Kimura N (2007) Mechanisms controlling the temporal variation of the sea ice edge in the Southern Ocean. J Oceanogr 63: 685–694.

[54] Arrigo KR, van Dijken GL (2003) Phytoplankton dynamics within 37 Antarctic coastal polynyas. J Geophys Res 108(C8): 3271 doi:10.1029/2002JC001739

[55] Rintoul SR (1998) On the origin and influence of Adélie Land BottomWater. In: Jacobs S, Weiss R, editors. Ocean, ice and the atmosphere: Interactions at the Antarctic continental margin. Antarctic Research Series 75. Washington DC: American Geophysical Union. pp. 151–171.

[56] Bindoff NL, Rintoul SR, Massom R (2000b) Bottom water formation and polynyas in Adélie Land, Antarctica. Papers Proc Roy Soc Tasmania 133(3): 51–56.

[57] Galton-Fenzi B (2009) Modelling ice-shelf/ocean interaction. Hobart, Tasmania: University of Tasmania. PhD Thesis.

[58] Kern S (2009) Wintertime Antarctic coastal polynya area: 1992–2008. Geophys Res Lett 36: L14501 doi:10.1029/2009GL038062

[59] Comiso JC, Gordon AL (1996) Cosmonaut polynya in the Southern Ocean: Structure and variability. J Geophys Res 101(C8): 18,297–18,313 doi:10.1029/96JC01500

[60] Arbetter TE, Lynch AH, Bailey DA (2004) Relationship between synoptic forcing and polynya formation in the Cosmonaut Sea: 1. Polynya climatology. J Geophys Res 109: C04022 doi:10.1029/2003JC001837

[61] Ohshima KI, Fukamachi Y, Williams GD, Nihashi S, Roquet F, et al (2013) Antarctic Bottom Water production by intense sea-ice formation in the Cape Darnley polynya. Nature Geosc 6(3): 235–240 doi:10.1038/ngeo1738

[62] Nihashi S, Ohshima KI, Jeffries MO, Kawamura T (2005) Sea-ice melting processes inferred from ice–upper ocean relationships in the Ross Sea, Antarctica. J Geophys Res 110: C02002 doi:10.1029/2003JC002235

[63] Nihashi S, Ohshima KI (2001) Relationship between the sea ice decay and solar heating through open water in the Antarctic sea ice zone. J Geophys Res 106: : 16,767–16,782.

[64] Holland PR, Kwok R (2012) Wind-driven trends in Antarctic sea-ice drift. Nature Geosc 5(11): 1–4 doi:10.1038/ngeo1627

[65] Massom RA, Comiso JC, Worby AP, Lytle VI, Stock L (1999) Satellite and in situ observations of regional classes of sea ice cover in the East Antarctic pack in winter. Rem Sens Envir 68(1): 61–76.

[66] Massom RA, Hill KL, Lytle VI, Worby AP, Paget MJ, et al (2001) Effects of regional fast-ice and iceberg distributions on the behaviour of the Mertz Glacier polynya, East Antarctica. Ann Glaciol 33: 391–398.

[67] Barber DG, Massom RA (2007) A bi-polar assessment of modes of polynya formation. In: Smith WO, Barber DG, editors. Polynyas: Windows to the World. Amsterdam: Elsevier, Amsterdam. pp. 1–54.

[68] Massom RA, Hill K, Barbraud C, Adams N, Ancel A, et al (2009) Fast ice distribution in Adélie Land, East Antarctica: Interannual variability and implications for Emperor penguins (*Aptenodytes forsteri*). Mar Ecol Progr Ser 374: 243–257.

[69] Smith MB, Labat J-P, Fraser AD, Massom RA, Koubbi P (2011) A GIS approach to estimating interannual variability of sea ice concentration in the Dumont d'Urville Sea near Terre Adélie from 2003 to 2009. Polar Science 5: 104–117.

[70] Tamura T, Williams GD, Fraser AD, Ohshima KI (2012), Potential regime shift in decreased sea ice production after the Mertz Glacier calving. Nature Comms: doi:10.1038/ncomms1820 10.1038/ncomms1820 22569370

[71] Wadhams P (2000) Ice in the Ocean. London: Gordon and Breech. 364 p.

[72] Rintoul SR, Sokolov S, Massom RA (2008) Rapid development and persistence of a massive Antarctic sea ice tongue. J Geophys Res 113: C07045 doi:10.1029/2007JC004541

[73] Massom RA, Stammerjohn SE, Smith RC, Pook MJ, Iannuzzi RA, et al (2006) Extreme anomalous atmospheric circulation in the West Antarctic Peninsula region in austral spring and summer 2001/02, and its profound impact on sea ice and biota. J Clim 19: 3544–3571.

[74] Massom RA, Stammerjohn S, Lefebvre W, Harangozo S, Adams N, et al (2008) West Antarctic Peninsula sea ice in 2005: Extreme ice compaction and ice edge retreat due to strong anomaly with respect to climate. J Geophys Res 113: C02S20 doi:10.1029/2007JC004239

